# APIM-guided dyadic nursing after colorectal cancer surgery: a propensity score-matched cohort study

**DOI:** 10.3389/fonc.2026.1909301

**Published:** 2026-09-03

**Authors:** Huili Du, Jing Wang

**Affiliations:** 1 Department of Surgical Oncology, Xinxiang Central Hospital, The Fourth Clinical College of Xinxiang Medical University, Xinxiang, Henan, China; 2 Department of Pharmacy, Xinxiang Central Hospital, The Fourth Clinical College of Xinxiang Medical University, Xinxiang, Henan, China

**Keywords:** actor-partner interdependence model, colorectal cancer, dyadic intervention, postoperative nursing, propensity score matching, self-efficacy

## Abstract

**Background:**

Recovery after colorectal cancer surgery is influenced not only by patient symptoms but also by caregiver capacity, psychological distress, self-efficacy, and dyadic coping. However, real-world evidence on Actor–Partner Interdependence Model (APIM)-guided dyadic nursing in routine postoperative care remains limited.

**Objective:**

To examine the association between documented exposure to APIM-guided dyadic nursing and 6-month postoperative outcomes among colorectal cancer patient–caregiver dyads, and to explore potential actor–partner pathways.

**Methods:**

This propensity score-matched retrospective cohort study reviewed postoperative colorectal cancer patient–caregiver dyads identified from electronic medical records, nursing documentation, and structured follow-up records. A total of 292 dyads were screened, and 252 dyads were retained after eligibility assessment and 1:1 propensity score matching, including 126 dyads exposed to APIM-guided dyadic nursing and 126 dyads receiving usual postoperative nursing. Outcomes were assessed at baseline/discharge and at 1, 3, and 6 months after discharge. Linear mixed-effects models estimated adjusted longitudinal associations. APIM-based generalized estimating equations and exploratory structural equation modeling were used to examine actor–partner associations and possible indirect pathways.

**Results:**

Compared with usual postoperative nursing, APIM-guided dyadic nursing exposure was associated with more favorable changes in patient colorectal cancer-specific quality of life, psychological distress, bowel dysfunction, self-efficacy, and dyadic coping. Caregivers in the APIM-guided nursing group also showed lower burden and distress, together with better quality of life and caregiving preparedness. Exploratory APIM analyses suggested stronger and more consistent actor associations than partner associations, with self-efficacy and distress emerging as key dyadic factors. Possible indirect pathways through patient self-efficacy, caregiver self-efficacy, and shared dyadic coping were also observed.

**Conclusions:**

In this propensity score-matched retrospective cohort study, APIM-guided dyadic nursing was associated with better postoperative patient and caregiver outcomes. These findings should be interpreted as adjusted associations rather than definitive causal evidence. Prospective multicenter randomized trials are needed to confirm effectiveness and implementation feasibility.

## 1 Introduction

Colorectal cancer is a major global health burden, accounting for approximately 1.9 million new cases and more than 900,000 deaths worldwide in 2022 (1–3). Its incidence among younger adults is also increasing in many countries, highlighting the growing need for effective postoperative recovery and survivorship care (4). Despite advances in enhanced recovery protocols and cancer survivorship services, patients undergoing colorectal cancer surgery commonly experience bowel dysfunction, fatigue, pain, nutritional problems, stoma-related difficulties, psychological distress, and challenges in adhering to long-term rehabilitation recommendations (5–7).

Postoperative recovery often occurs within a family caregiving context. Family caregivers assist with symptom monitoring, medication administration, diet, mobility, wound or stoma care, emotional support, and recognition of potential complications (8, 9). However, caregivers frequently receive limited preparation and may themselves experience psychological distress, unmet supportive care needs, and substantial caregiving burden (10, 11). Conventional discharge nursing primarily focuses on the patient and may therefore inadequately address the reciprocal influence between patients and caregivers during home recovery.

Patient and caregiver outcomes are increasingly understood as interdependent (9, 12). Self-efficacy, psychological distress, dyadic coping, caregiver burden, and caregiving preparedness may affect not only an individual’s own outcomes but also those of the other member of the dyad (13, 14). Compared with self-efficacy-focused care models, Actor–Partner Interdependence Model (APIM) captures both individual and reciprocal patient–caregiver effects, allowing postoperative nursing to address the dyad rather than either member alone (15). Nevertheless, APIM has rarely been incorporated into structured postoperative nursing for colorectal cancer patient–caregiver dyads, and real-world evidence regarding its clinical application remains limited.

Therefore, this propensity score-matched retrospective cohort study examined the association between documented exposure to APIM-guided dyadic nursing and 6-month postoperative outcomes among colorectal cancer patient–caregiver dyads. We evaluated patient health-related quality of life, psychological distress, bowel function, self-efficacy, and dyadic coping, as well as caregiver burden, distress, quality of life, preparedness, and self-efficacy. We also explored potential actor–partner associations and indirect pathways involving patient self-efficacy, caregiver self-efficacy, and shared dyadic coping.

## 2 Methods

### 2.1 Study design, setting, and ethical approval

This retrospective cohort study was conducted using routinely collected clinical records, nursing documentation, and structured postoperative follow-up data from Xinxiang Central Hospital. Patient–caregiver dyads who underwent radical surgery for colorectal cancer and had eligible routine postoperative nursing and follow-up records during the study period were screened retrospectively. Dyads were classified into the APIM-guided dyadic nursing group or the usual postoperative nursing group according to documented postoperative nursing exposure. Baseline was defined as the discharge assessment or the first documented postoperative nursing assessment before home-based recovery. Follow-up assessments were extracted at 1, 3, and 6 months after discharge when available.

Due to the use of retrospective clinical and nursing records in this study, approval was obtained from the Ethics Committee of Xinxiang Central Hospital (Ethics Approval Number: 20260612). All extracted data is anonymous before analysis to protect the confidentiality of participants.

### 2.2 Study population and exposure classification

A total of 292 postoperative colorectal cancer patient–caregiver dyads were screened from the hospital information system and nursing follow-up database. After excluding 40 dyads because of incomplete eligibility information, absence of an identifiable family caregiver, severe cognitive impairment or major psychiatric crisis, discharge to institutional care, expected survival less than 6 months, or insufficient baseline/follow-up data, 252 eligible dyads entered propensity score estimation. Exposure status was determined from nursing records and classified as APIM-guided dyadic nursing or usual postoperative nursing. Propensity scores were estimated using baseline patient, caregiver, tumor, surgical, and psychosocial variables. Eligible dyads were matched 1:1 using nearest-neighbor matching without replacement within the prespecified caliper. All matched pairs met the caliper criterion, and the final analytic cohort included 252 dyads, with 126 dyads in each nursing exposure group.

### 2.3 Exposure: APIM-guided dyadic nursing

**Figure 1:**
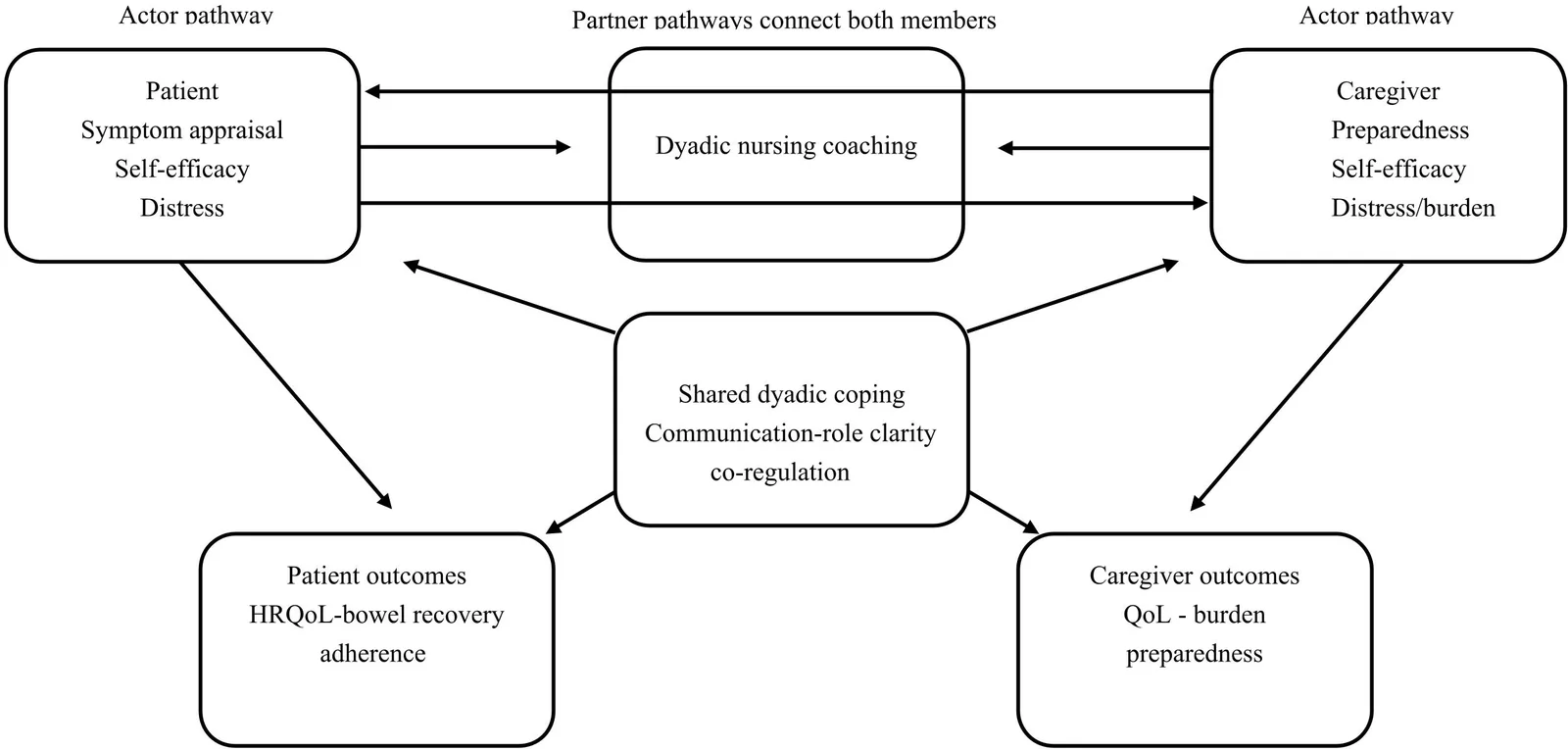
Theoretical mechanism framework linking dyadic nursing, APIM pathways, and postoperative recovery outcomes.

The APIM-informed conceptual framework linking documented dyadic nursing exposure, actor–partner pathways, shared dyadic coping, and postoperative patient and caregiver outcomes is shown in **Figure 1**.

#### 2.3.1 Intervention rationale and materials

The APIM-guided dyadic nursing program treated the patient–caregiver dyad as the unit of care. Its rationale was based on the Actor–Partner Interdependence Model, which proposes that the psychological and behavioral characteristics of each dyad member may influence both their own outcomes and those of their partner. The program therefore targeted patient and caregiver self-efficacy, psychological distress, symptom-management behaviors, communication, dyadic coping, caregiving preparedness, and task sharing. Intervention delivery was supported by a standardized nursing manual and structured materials, including a dyadic assessment form, a postoperative symptom co-monitoring checklist, teach-back prompts, rehabilitation goal-setting forms, a communication and emotional validation guide, a caregiving task-sharing worksheet, caregiver burden screening items, a respite-planning form, warning-sign escalation criteria, and a structured follow-up documentation template.

#### 2.3.2 Intervention providers and nurse training

The program was delivered by 3 registered nurses working in the surgical oncology department. The nurses had at least 5 years of experience in surgical or oncology nursing. Before delivering the program, they completed a standardized 72-hour training program covering the principles of APIM, dyadic needs assessment, postoperative symptom management, teach-back methods, self-efficacy enhancement, communication and emotional co-regulation, caregiver burden assessment, escalation criteria, and standardized intervention documentation. Training included didactic instruction, review of the intervention manual, case-based discussion, and simulated patient–caregiver sessions. Nurse competence was assessed using [A WRITTEN TEST/A SIMULATED SESSION/A MODULE CHECKLIST], and nurses were required to achieve 80% of the predefined competency criteria before independently delivering the program. Refresher discussions were conducted to address implementation questions and maintain consistency.

#### 2.3.3 Delivery mode, setting, schedule, and duration

The intervention began during discharge preparation in the surgical ward. A structured face-to-face session was conducted jointly with the patient and primary family caregiver in a consultation area or at the bedside when privacy and the patient’s clinical condition permitted. The initial session lasted approximately 60–120 minutes. The session addressed six core modules: dyadic formulation, symptom co-monitoring, self-efficacy rehearsal, communication and emotional co-regulation, caregiving load sharing and respite planning, and transition and relapse prevention. Skills involving wound or stoma care, diet progression, medication use, ambulation, and recognition of warning signs were demonstrated and assessed using teach-back or return demonstration where applicable.

After discharge, structured follow-up contacts were scheduled at weeks 2, 4, 8, 12, and 24. Follow-up was delivered by telephone or the hospital-approved digital communication platform and generally lasted approximately [NUMBER–NUMBER] minutes per contact. During each contact, the nurse reviewed symptoms, rehabilitation progress, care-task allocation, patient and caregiver distress, self-efficacy, adherence barriers, and the need for escalation or referral. Additional contact could occur when serious symptoms, substantial psychological distress, high caregiver burden, or difficulties performing essential care tasks were documented.

#### 2.3.4 Standardization and permitted tailoring

The six core modules, their intended sequence, the scheduled follow-up points, and the required documentation fields were standardized across dyads (**Table 1**). Nurses used the same intervention manual, module checklists, teach-back prompts, symptom-monitoring domains, warning-sign criteria, and follow-up record template. Tailoring was permitted within the standardized framework. Nurses could adjust the emphasis and time allocated to individual modules according to cancer site, stoma status, symptom burden, functional ability, psychological distress, caregiver preparedness, family resources, and the division of caregiving tasks. Individual rehabilitation goals and escalation plans were personalized, but the core intervention components were not omitted unless they were clinically inapplicable, in which case the reason was documented.

**Table 1:** Operational definition and documented components of APIM-guided dyadic nursing exposure.

Component	Core nursing activities	APIM mechanism
Module 1: Dyadic formulation	Nurse maps symptom load, caregiving tasks, fears, and relationship resources for each dyad.	Shared problem representation; identification of actor and partner targets.
Module 2: Symptom co-monitoring	Patient and caregiver use a daily bowel, pain, fatigue, nutrition, wound/stoma and warning-sign checklist.	Moves caregiver support from vigilance alone to calibrated partner responsiveness.
Module 3: Self-efficacy rehearsal	Teach-back of stoma care, ambulation, diet progression, medication and complication response.	Strengthens patient actor efficacy and caregiver partner efficacy.
Module 4: Communication and emotional co-regulation	Structured 10-minute dyadic conversation, emotion labeling, validation, and escalation plan.	Reduces distress contagion and promotes adaptive dyadic coping.
Module 5: Load-sharing and respite	Care-task negotiation, caregiver burden screening, micro-respite prescription, referral triggers.	Protects caregiver functioning and reduces overprotection.
Module 6: Transition and relapse prevention	Personalized rehabilitation goals, follow-up schedule, and digital nurse contact at weeks 2, 4, 8, 12, and 24.	Sustains rehabilitation adherence after discharge.

#### 2.3.5 Adherence and intervention fidelity

Because this was a retrospective cohort study, intervention adherence and fidelity were evaluated from existing nursing records rather than through prospective direct observation. A planned intervention component was classified as completed only when the corresponding required activities were documented in the structured nursing record. Follow-up adherence was determined from the presence and content of records corresponding to the five scheduled post-discharge contacts. For each dyad, adherence was calculated as the number of documented completed intervention components divided by the total number of planned applicable components, expressed as a percentage. The primary exposure classification was based on documented receipt of the structured APIM-guided dyadic nursing program. In the adherence-based sensitivity analysis, high adherence was defined as documented completion of at least 70% of the planned applicable components.

### 2.4 Usual postoperative nursing

Usual postoperative nursing consisted of routine discharge education, wound and stoma care instructions when applicable, medication guidance, diet and activity advice, outpatient follow-up reminders, and *ad hoc* telephone consultation. Caregivers could attend routine education sessions, but they were not systematically assessed, coached, or assigned APIM-based actor–partner intervention targets. Usual care exposure was confirmed through routine discharge and nursing follow-up records.

### 2.5 Outcomes and measurement indicators

The primary patient outcome was health-related quality of life, measured by the FACT-C total score, with higher scores indicating better colorectal cancer-specific quality of life. Secondary patient outcomes included psychological distress measured by the HADS total score, bowel dysfunction measured using a low-anterior-resection-syndrome-style score, patient self-efficacy, and shared dyadic coping. Caregiver outcomes included Zarit caregiver burden, caregiver HADS total score, caregiver quality of life, preparedness for caregiving, and caregiver self-efficacy. Secondary clinical outcomes included documented postoperative complications and unplanned readmission within 30 days after discharge, identified from electronic medical records and follow-up documentation.

All outcomes were extracted from structured nursing assessment forms, postoperative follow-up records, and electronic medical records. Only outcomes documented as part of routine clinical or nursing follow-up were included in the analysis. When more than one record was available within a follow-up window, the record closest to the planned assessment time point was used.

### 2.6 Propensity score matching and covariate balance

Because treatment exposure was not randomly assigned, propensity score matching was used to reduce baseline imbalance and selection bias. Propensity scores were estimated using logistic regression, with receipt of APIM-guided dyadic nursing as the dependent variable. Covariates included patient age, sex, cancer site, pathological stage, stoma status, surgical approach, baseline FACT-C score, baseline HADS score, baseline bowel dysfunction score, caregiver age, caregiver sex, spousal caregiver status, education level, baseline caregiver burden, caregiver quality of life, patient self-efficacy, caregiver self-efficacy, and dyadic coping.

Dyads were matched 1:1 using nearest-neighbor matching without replacement, with a caliper of 0.2 standard deviations of the logit of the propensity score. Baseline balance before and after matching was assessed using standardized mean differences. An absolute standardized mean difference less than 0.10 was considered acceptable balance.

### 2.7 Statistical analysis

Continuous variables were summarized as mean ± standard deviation or median with interquartile range, as appropriate, and categorical variables as number and percentage. Baseline covariate balance after propensity score matching was assessed using standardized mean differences, with an absolute value <0.10 indicating acceptable balance. Longitudinal outcomes were analyzed using linear mixed-effects models with fixed effects for nursing group, categorical time, and the group-by-time interaction, and a random intercept for each dyad. The 6-month interaction coefficient represented the adjusted between-group difference in change from baseline. Standardized effect sizes were calculated by dividing the adjusted estimate and its confidence limits by the pooled baseline standard deviation of the corresponding outcome. Missing repeated observations were handled under the likelihood-based missing-at-random assumption.

APIM analyses were conducted using generalized estimating equations with an identity link, exchangeable working correlation structure, and robust standard errors to account for within-dyad dependence. Continuous predictors were standardized, and linearity, influential observations, and multicollinearity were assessed. Exploratory mediation was examined using structural equation modeling with robust maximum-likelihood estimation and 5,000 bootstrap samples for indirect-effect confidence intervals. Model fit was evaluated using CFI, TLI, RMSEA, and SRMR, with CFI/TLI ≥0.90 and RMSEA/SRMR ≤0.08 indicating acceptable fit.

FACT-C was the prespecified primary outcome and was tested at a two-sided significance level of 0.05. For secondary patient and caregiver outcomes, the Benjamini–Hochberg procedure controlled the false discovery rate at 5%. APIM and indirect-effect analyses were exploratory and were interpreted primarily using effect estimates and 95% confidence intervals. Mixed-effects models included all available repeated measurements under the missing-at-random assumption, without single-value imputation. Dyads lacking sufficient baseline information for propensity score estimation were excluded before matching. Structural equation models used full-information maximum likelihood where applicable, and complete-case analyses were performed as sensitivity analyses. Mixed-effects models used all available repeated measurements under the missing-at-random assumption, whereby missingness was assumed to depend on observed variables but not on unobserved outcomes after covariate adjustment. No single-value imputation was performed, and complete-case analyses were used as sensitivity analyses.

Intraclass correlation coefficients and variance components were reported for the principal mixed-effects models. The GEE-based APIM and SEM mediation analyses were prespecified as exploratory secondary analyses and were interpreted as hypothesis-generating rather than confirmatory. Because of the retrospective observational design, the indirect-effect analysis was considered exploratory and hypothesis-generating and was not interpreted as evidence of causal mediation. Sensitivity analyses included complete-case ANCOVA, inverse-probability weighted analysis, and an adherence-based subgroup analysis among dyads completing at least 70% of planned intervention components. All tests were two-sided, and P <0.05 was considered statistically significant.

## 3 Results

### 3.1 Cohort selection and baseline characteristics after propensity score matching

**Figure 2:**
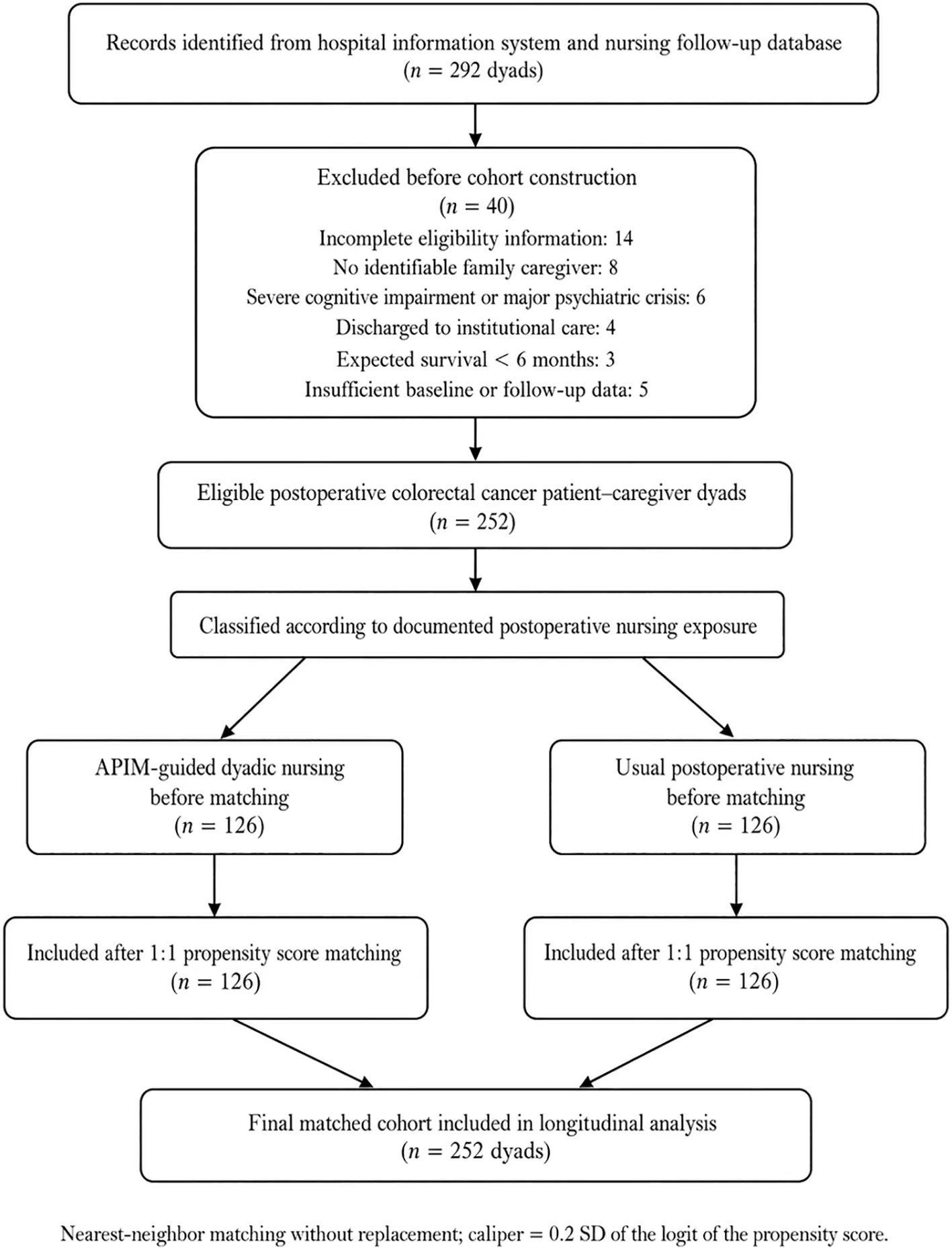
Flow diagram of retrospective cohort selection and propensity score matching.

**Figure 2** shows the retrospective cohort selection and propensity score matching process. A total of 292 postoperative colorectal cancer patient–caregiver dyads were identified from the hospital information system and nursing follow-up database. After exclusion of 40 dyads because of incomplete eligibility information, absence of an identifiable family caregiver, severe cognitive impairment or major psychiatric crisis, discharge to institutional care, expected survival less than 6 months, or insufficient baseline/follow-up data, 252 eligible dyads were retained for cohort construction. These dyads were classified according to documented postoperative nursing exposure. After 1:1 propensity score matching, the final matched cohort included 126 dyads in the APIM-guided dyadic nursing group and 126 dyads in the usual postoperative nursing group.

**Table 2** presents the baseline characteristics of the propensity score-matched cohort. After 1:1 propensity score matching, 252 patient–caregiver dyads were retained for analysis, including 126 dyads in the APIM-guided dyadic nursing group and 126 dyads in the usual postoperative nursing group. Patient age was similar between groups, with a mean age of 61.4 years in the APIM-guided dyadic nursing group and 62.2 years in the usual care group. Baseline scores for patient FACT-C, patient HADS, bowel dysfunction, caregiver burden, caregiver quality of life, patient self-efficacy, and caregiver self-efficacy were also comparable between groups, with most standardized mean differences below 0.10. Some variables, including patient sex, rectal cancer, laparoscopic surgery, dyadic coping, and spousal caregiver status, showed mild residual imbalance. Therefore, these factors were further considered in adjusted longitudinal and sensitivity analyses. Overall, the matched cohort showed acceptable baseline comparability for the main patient- and caregiver-reported outcomes.

**Table 2:** Baseline characteristics of the propensity score-matched postoperative colorectal cancer patient–caregiver dyads.

Characteristic	APIM dyadic nursing (n=126)	Usual care (n=126)	SMD
Patient age, years	61.4 ± 9.2	62.2 ± 9.6	-0.08
Caregiver age, years	56.3 ± 15.5	57.2 ± 15.6	-0.06
Patient FACT-C total	84.1 ± 15.1	83.9 ± 14.2	0.01
Patient HADS total	17.4 ± 7.2	17.7 ± 6.4	-0.04
Patient bowel dysfunction (LARS)	25.4 ± 7.5	26.4 ± 7.4	-0.13
Caregiver Zarit burden	30.8 ± 10.2	30.3 ± 11.1	0.05
Caregiver quality of life	66.1 ± 11.7	66.7 ± 12.1	-0.05
Patient self-efficacy	53.6 ± 11.8	54.3 ± 12.1	-0.06
Caregiver self-efficacy	52.0 ± 11.0	51.0 ± 11.7	0.09
Dyadic coping	54.5 ± 12.2	52.5 ± 13.4	0.16
Patient male sex	71 (56.3)	58 (46.0)	0.21
Caregiver female sex	76 (60.3)	76 (60.3)	0.0
Spousal caregiver	86 (68.3)	76 (60.3)	0.17
College education or higher	58 (46.0)	52 (41.3)	0.1
Temporary/permanent stoma	31 (24.6)	38 (30.2)	-0.12
Rectal cancer	57 (45.2)	71 (56.3)	-0.22
Laparoscopic surgery	96 (76.2)	83 (65.9)	0.23
Pathological stage I	24 (19.0)	19 (15.1)	0.11
Pathological stage II	47 (37.3)	46 (36.5)	0.02
Pathological stage III	55 (43.7)	61 (48.4)	-0.1

Data are presented as mean ± SD or n (%). SMD, standardized mean difference. Absolute SMD values <0.10 indicate good balance. Variables with residual imbalance were further adjusted in longitudinal models.

### 3.2 Longitudinal associations with patient and caregiver outcomes

**Table 3** presents the longitudinal patient and caregiver outcomes in the propensity score-matched cohort. The estimated ICCs indicated moderate within-dyad clustering, ranging from 0.36 to 0.44 across the principal outcomes. For the FACT-C model, the random-intercept variance was 68.40 and the residual variance was 92.60; the corresponding values for the patient HADS model were 8.70 and 15.40, respectively. Compared with usual postoperative nursing, APIM-guided dyadic nursing exposure was associated with more favorable 6-month changes in both patient and caregiver outcomes. For patients, the FACT-C total score increased more markedly in the APIM-guided nursing group, with an adjusted between-group difference in change of 10.49 points. Psychological distress and bowel dysfunction also showed greater reductions, with adjusted differences of -3.91 and -4.77 points, respectively. Patient self-efficacy and dyadic coping improved more in the APIM-guided nursing group, with adjusted differences of 9.45 and 8.96 points.

**Table 3:** Longitudinal patient and caregiver outcomes in the propensity score-matched cohort.

Outcome	APIM-guided nursing baseline	Usual care baseline		APIM-guided nursing 6 months	Usual care 6 months		Adjusted between-group difference in change at 6 months	Standardized effect size (95% CI)	P
Patient FACT-C total	84.1 ± 15.1	83.9 ± 14.2	104.4 ± 15.3			93.8 ± 14.2	10.49 (8.37 to 12.62)	0.72 (0.57 to 0.86)	<0.001
Patient HADS total	17.4 ± 7.2	17.7 ± 6.4	9.5 ± 6.1			13.7 ± 6.1	-3.91 (-4.96 to -2.86)	−0.57 (−0.73 to −0.42)	<0.001
Patient bowel dysfunction (LARS)	25.4 ± 7.5	26.4 ± 7.4	16.3 ± 6.8			22.1 ± 7.4	-4.77 (-6.06 to -3.48)	−0.64 (−0.81 to −0.47)	<0.001
Patient self-efficacy	53.6 ± 11.8	54.3 ± 12.1	70.5 ± 12.9			61.4 ± 12.5	9.45 (7.87 to 11.03)	0.79 (0.66 to 0.92)	<0.001
Dyadic coping	54.5 ± 12.2	52.5 ± 13.4	72.2 ± 12.3			61.2 ± 13.7	8.96 (7.16 to 10.75)	0.70 (0.56 to 0.84)	<0.001
Caregiver Zarit burden	30.8 ± 10.2	30.3 ± 11.1	18.1 ± 9.9			23.9 ± 11.3	-6.31 (-7.87 to -4.75)	−0.59 (−0.74 to −0.45)	<0.001
Caregiver HADS total	15.9 ± 6.1	16.5 ± 6.0	8.9 ± 5.7			13.0 ± 6.0	-3.42 (-4.54 to -2.29)	−0.57 (−0.75 to −0.38)	<0.001
Caregiver quality of life	66.1 ± 11.7	66.7 ± 12.1	81.3 ± 11.6			75.1 ± 12.5	6.71 (4.74 to 8.68)	0.56 (0.40 to 0.73)	<0.001
Caregiver preparedness	18.6 ± 5.0	18.9 ± 4.9	25.3 ± 4.7			21.7 ± 5.0	4.02 (3.20 to 4.84)	0.81 (0.65 to 0.98)	<0.001

At 6 months, outcome data were available for 124 of 126 dyads in the APIM-guided nursing group and 122 of 126 dyads in the usual care group. The six missing assessments resulted from unsuccessful contact (n = 3), missed follow-up visits (n = 2), and incomplete outcome documentation (n = 1).

Similar favorable associations were observed among caregivers. APIM-guided dyadic nursing was associated with a greater reduction in caregiver burden and distress, with adjusted differences of -6.31 and -3.42 points, respectively. Caregiver quality of life and preparedness also improved more in the APIM-guided nursing group. These findings suggest that documented exposure to APIM-guided dyadic nursing was associated with better postoperative recovery and caregiver well-being in the matched retrospective cohort.

**Figure 3:**
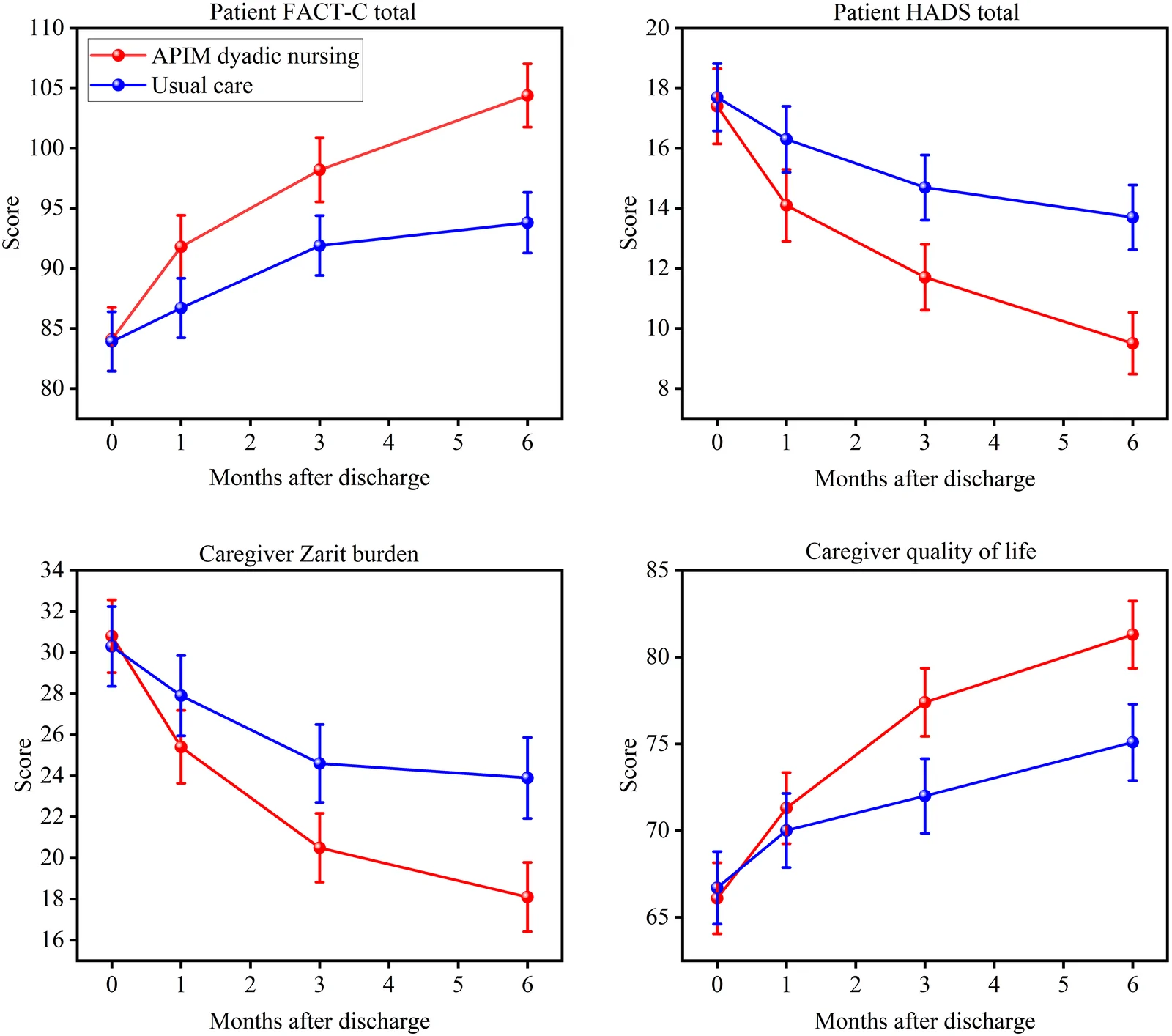
Longitudinal trajectories of patient and caregiver outcomes. Error bars represent 95% confidence intervals of observed means.

**Figure 3** shows the longitudinal trajectories of four key patient and caregiver outcomes from baseline to 6 months after discharge. At baseline, the two matched groups had generally similar scores. Over time, the APIM-guided dyadic nursing group showed more favorable trajectories than the usual care group. Patient FACT-C total score and caregiver quality of life increased more markedly in the APIM-guided nursing group, whereas patient HADS total score and caregiver Zarit burden decreased more clearly. The between-group separation became visible from 1 to 3 months and was maintained at 6 months. Postoperative complications occurred in 14 patients (11.1%) in the APIM-guided nursing group and 22 patients (17.5%) in the usual care group. Unplanned 30-day readmission occurred in 6 patients (4.8%) and 11 patients (8.7%), respectively.

### 3.3 Exploratory actor–partner interdependence associations

**Figure 4:**
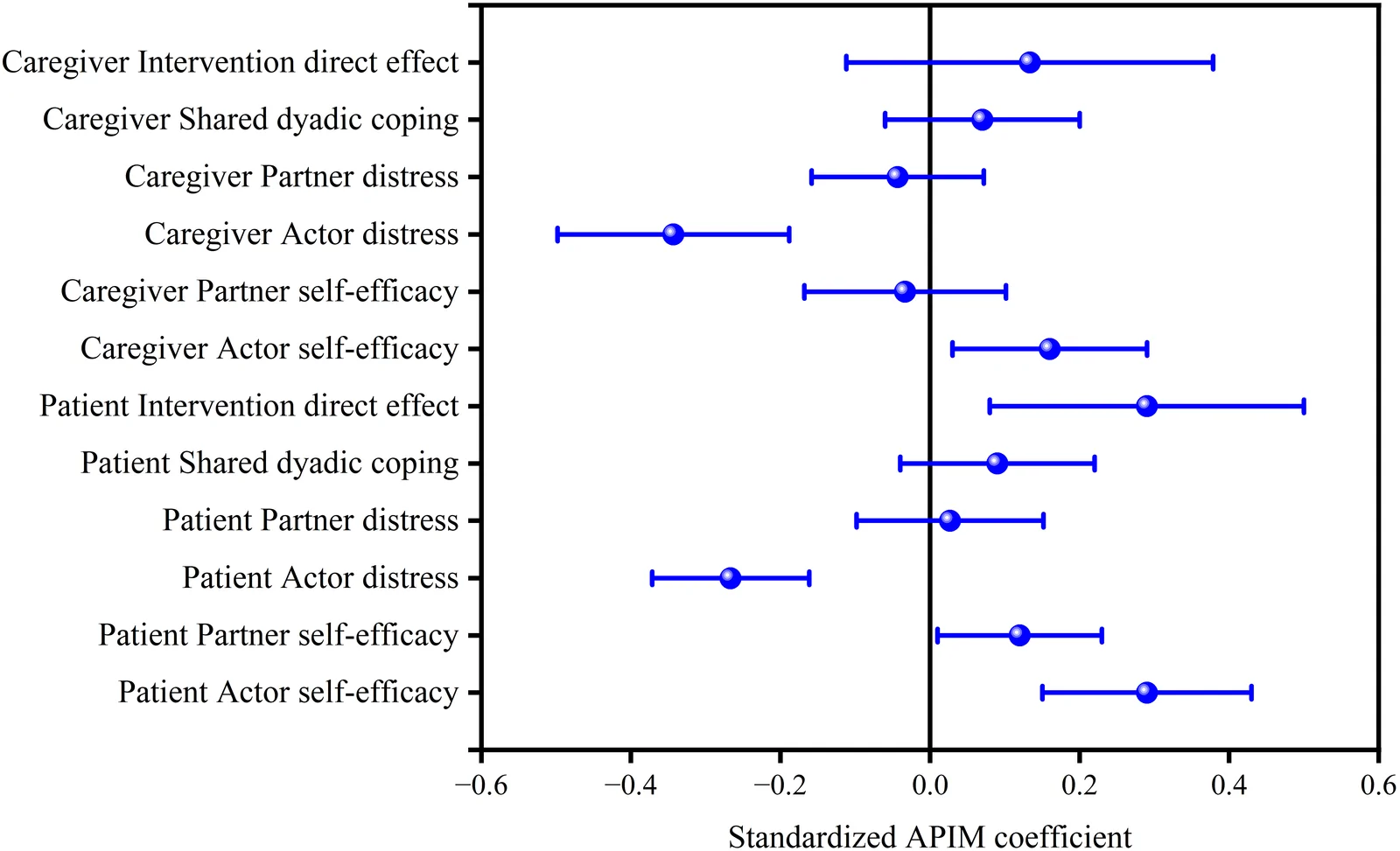
Exploratory forest plot of role-specific actor and partner associations from APIM models.

Exploratory APIM analyses showed that actor effects were stronger and more consistent than partner associations. For patient outcomes, higher actor self-efficacy was associated with better adaptive functioning (β = 0.29, 95% CI: 0.15 to 0.43), whereas higher actor distress was associated with poorer functioning (β = -0.27, 95% CI: -0.37 to -0.16). Caregiver self-efficacy showed a modest partner association with patient recovery (β = 0.12, 95% CI: 0.01 to 0.23). For caregiver outcomes, self-efficacy was positively associated with functioning, while distress showed the strongest negative association. **Figure 4**.

### 3.4 Exploratory indirect-effect and moderation analyses

**Figure 5:**
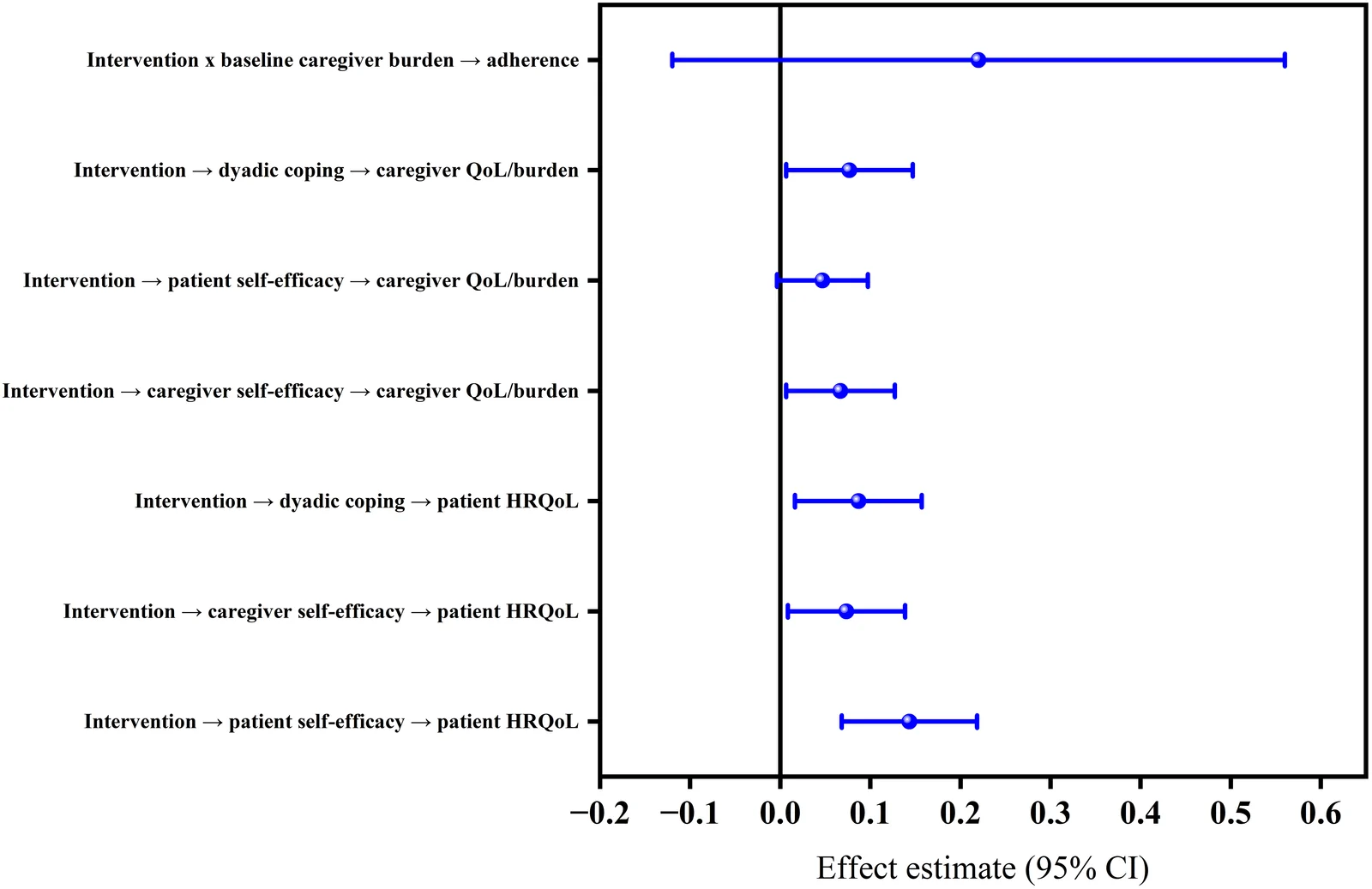
Exploratory forest plot of APIM-informed indirect-effect and moderation estimates.

Exploratory mediation and moderation analyses were conducted to examine possible indirect pathways linking APIM-guided dyadic nursing exposure to 6-month outcomes. **Figure 5** presents the APIM-informed mediation and moderation estimates. For patient health-related quality of life, a statistically significant indirect association through patient self-efficacy was observed and showed the largest estimate (estimate = 0.14, 95% CI: 0.07 to 0.22). Indirect pathways through caregiver self-efficacy (estimate = 0.07, 95% CI: 0.01 to 0.14) and shared dyadic coping (estimate = 0.08, 95% CI: 0.02 to 0.16) were also statistically significant.

For caregiver outcomes, APIM-guided dyadic nursing exposure showed significant indirect associations through caregiver self-efficacy (estimate = 0.06, 95% CI: 0.01 to 0.13) and shared dyadic coping (estimate = 0.07, 95% CI: 0.01 to 0.15). The pathway from patient self-efficacy to caregiver quality of life or burden was not clearly significant (estimate = 0.04, 95% CI: 0.00 to 0.10). The moderation term for APIM-guided nursing exposure × baseline caregiver burden on rehabilitation adherence was also not statistically significant (estimate = 0.22, 95% CI: -0.12 to 0.56). These indirect-effect estimates represent exploratory statistical associations consistent with possible pathways involving patient self-efficacy, caregiver self-efficacy, and shared dyadic coping. They should not be interpreted as evidence that these variables causally mediated the association between APIM-guided dyadic nursing exposure and postoperative outcomes. The moderation analysis was also exploratory and did not identify a statistically significant interaction between nursing exposure and baseline caregiver burden.

### 3.5 Sensitivity analyses

Sensitivity analyses were conducted to examine the robustness of the main 6-month findings. Across complete-case ANCOVA, inverse-probability weighted analysis, and adherence-based subgroup analysis among dyads with documented completion of at least 70% of planned APIM-guided nursing modules, the results remained consistent in direction and magnitude.

For patient FACT-C total score, the estimated associations ranged from 8.35 to 8.96 points, and all confidence intervals excluded zero. For patient HADS total score, the estimates ranged from -3.43 to -3.36 points, suggesting a consistent association with lower psychological distress. Similar patterns were observed for caregiver outcomes. The estimated reduction in caregiver Zarit burden ranged from -5.22 to -5.05 points, while the improvement in caregiver quality of life ranged from 5.96 to 6.64 points. All sensitivity analyses showed statistically significant results, with P values less than 0.001. These findings suggest that the observed associations between APIM-guided dyadic nursing and patient-caregiver outcomes were not dependent on a single analytic approach. **Table 4**.

**Table 4:** Sensitivity analyses for selected 6-month outcomes.

Outcome	Analytical method	Adjusted between-group difference at 6 months (95% CI)	Standardized effect size (95% CI)	P
Patient FACT-C total	Complete-case ANCOVA at 6 months	8.35 (6.75 to 9.94)	0.57 (0.46 to 0.68)	<0.001
Patient FACT-C total	Inverse-probability weighted ANCOVA	8.21 (6.66 to 9.75)	0.55 (0.43 to 0.67)	<0.001
Patient FACT-C total	Adherence-based subgroup analysis, module completion ≥70%	8.96 (7.20 to 10.71)	0.61 (0.49 to 0.73)	<0.001
Patient HADS total	Complete-case ANCOVA at 6 months	-3.43 (-4.13 to -2.74)	−0.49 (−0.59 to −0.40)	<0.001
Patient HADS total	Inverse-probability weighted ANCOVA	-3.50 (-4.25 to -2.75)	−0.51 (−0.62 to −0.40)	<0.001
Patient HADS total	Adherence-based subgroup analysis, module completion ≥70%	-3.36 (-4.19 to -2.53)	−0.48 (−0.60 to −0.36)	<0.001
Caregiver Zarit burden	Complete-case ANCOVA at 6 months	-5.22 (-6.34 to -4.09)	−0.46 (−0.52 to −0.38)	<0.001
Caregiver Zarit burden	Inverse-probability weighted ANCOVA	-5.31 (-6.44 to -4.19)	−0.50 (−0.60 to −0.39)	<0.001
Caregiver Zarit burden	Adherence-based subgroup analysis, module completion ≥70%	-5.05 (-6.32 to -3.78)	−0.47 (−0.59 to −0.35)	<0.001
Caregiver QoL	Complete-case ANCOVA at 6 months	5.96 (4.51 to 7.41)	0.55 (0.48 to 0.62)	<0.001
Caregiver QoL	Inverse-probability weighted ANCOVA	6.00 (4.55 to 7.45)	0.50 (0.38 to 0.63)	<0.001
Caregiver QoL	Adherence-based subgroup analysis, module completion ≥70%	6.64 (4.97 to 8.31)	0.56 (0.42 to 0.70)	<0.001

## 4 Discussion

### 4.1 Main findings and importance of this study

This propensity score-matched retrospective cohort study examined the association between APIM-guided dyadic nursing and postoperative recovery among colorectal cancer patient–caregiver dyads. In the matched cohort, APIM-guided dyadic nursing was associated with more favorable changes in patient health-related quality of life, psychological distress, bowel dysfunction, self-efficacy, and dyadic coping compared with usual postoperative nursing. Caregivers in the APIM-guided dyadic nursing group also showed lower burden and distress, as well as better quality of life and caregiving preparedness. These findings are consistent with recent dyadic evidence showing that caregiver burden, patient self-perceived burden, dyadic coping, anxiety, and depression are interrelated in colorectal cancer patient–spousal caregiver dyads (13).

The present study contributes to the existing dyadic care literature by examining an APIM-informed nursing approach in routine postoperative colorectal cancer care. As discussed in detail in Section 4.2, the present study extends the existing dyadic care literature by applying APIM as an organizing framework for a structured postoperative nursing approach, rather than using APIM solely to describe psychosocial interdependence between patients and caregivers. Instead of treating patients and caregivers as separate individuals, this model considers the dyad as an interdependent care unit. This dyadic perspective is supported by recent APIM-based evidence showing that perceived stress, relationship satisfaction, and distress disclosure may jointly shape emotional distress within colorectal cancer patient–caregiver dyads (13, 14, 16). Taken together, these studies indicate that psychological adjustment and coping during colorectal cancer care are shaped not only by the characteristics of each individual but also by reciprocal processes between patients and caregivers. The exploratory APIM findings suggested that actor effects were stronger and more consistent than partner effects. In particular, patient and caregiver self-efficacy were positively associated with adaptive functioning, whereas distress was negatively associated with recovery-related outcomes. A smaller caregiver-to-patient partner association was also observed, suggesting that caregiver confidence may be linked to better patient recovery. This finding is also consistent with dyadic ostomy-care research indicating that patient and caregiver psychological status can influence patient self-care and caregiver contribution to self-care (17). However, because this was a retrospective observational study, these findings should be interpreted as adjusted associations rather than definitive causal effects.

### 4.2 Comparison with previous studies

Self-efficacy interventions have improved confidence and several outcomes in colorectal cancer care. However, few studies have treated patients and caregivers as an interdependent care unit. Previous APIM studies mainly examined psychosocial interdependence within colorectal cancer dyads. Chen et al. linked dyadic coping with patient burden, caregiver burden, anxiety, and depression (13). Qin et al. identified actor and partner associations between family resilience and dyadic coping (14). Kim et al. reported interdependence between patient and caregiver health outcomes (15). Jin et al. found actor and partner pathways involving stress, disclosure, and emotional distress (16). Iovino et al. also reported reciprocal psychological influences on ostomy self-care (18). Our findings are consistent with these studies, particularly the strong actor effects of self-efficacy and distress. The caregiver-to-patient association also supports reciprocal influences during postoperative recovery. However, most previous APIM studies were cross-sectional and focused on explaining psychosocial relationships. Our study extended this work by applying APIM to structured nursing and examining longitudinal outcomes. The program also addressed communication, task sharing, caregiver burden, and shared coping. These components may explain the favorable changes observed in both dyad members.

### 4.3 Clinical implications

These findings have practical implications for postoperative colorectal cancer nursing. First, discharge nursing may benefit from shifting from a patient-only model to a dyadic care model. Family caregivers are not only informal assistants but also important participants in symptom monitoring, diet and activity support, stoma or wound care, emotional support, and complication recognition. Therefore, routine nursing assessment should include both patient needs and caregiver preparedness. Second, self-efficacy may be an important target for postoperative rehabilitation. Teach-back, symptom co-monitoring, goal setting, rehearsal of care tasks, and structured follow-up may help strengthen both patient and caregiver confidence. Third, dyadic coping and communication may be incorporated into discharge education. Structured communication, emotional validation, and shared problem-solving may help reduce distress and improve cooperation during home recovery. Finally, APIM-based assessment may help nurses identify high-risk dyads, such as patients with high distress paired with caregivers with low self-efficacy or high burden. These dyads may require earlier follow-up, more intensive telenursing, respite support, or referral to psycho-oncology services.

### 4.4 Limitations

Several limitations should be acknowledged. First, this was a retrospective cohort study rather than a randomized trial. Although propensity score matching and adjusted analyses were used, selection bias and residual confounding from unmeasured factors could not be fully excluded. Second, exposure classification depended on nursing documentation, so incomplete or inconsistent records may have caused misclassification of APIM-guided dyadic nursing or module completion. Third, several outcomes were extracted from routine follow-up records and self-reported scales, which may be affected by recall bias, social desirability bias, and variation in assessment timing. Fourth, some baseline variables still showed mild residual imbalance after matching, although they were further adjusted in outcome models. Finally, the indirect-effect analyses should not be interpreted as establishing causal mediation. The retrospective design did not permit definitive confirmation of temporal ordering between exposure, potential mediators, and outcomes, and unmeasured mediator–outcome confounding could not be excluded. These findings therefore indicate hypothesis-generating statistical pathways that require confirmation in prospective studies with repeated measurements.

### 4.5 Future research

Future studies should validate this APIM-guided dyadic nursing model in larger, prospective, multicenter studies. Randomized controlled trials with standardized intervention manuals, predefined fidelity assessment, and longer follow-up are needed to confirm whether APIM-guided dyadic nursing causally improves patient recovery and caregiver well-being. Future research should also evaluate implementation outcomes, including nurse workload, intervention fidelity, cost-effectiveness, digital follow-up feasibility, and acceptability among patients and caregivers. Future implementation studies may also consider digital delivery models, as recent randomized evidence has examined mobile application-based postoperative lifestyle management among patients after colorectal cancer surgery, although effects on quality of life and adherence require further confirmation (19).

Further studies should refine the intervention for different risk groups. Dyads with stoma care needs, high caregiver burden, low self-efficacy, high psychological distress, or poor family communication may require more intensive or tailored support. Different delivery formats, such as face-to-face nursing, telephone follow-up, mobile health platforms, or hybrid models, should also be compared. In addition, future APIM-based studies should collect repeated patient and caregiver psychosocial measures over time to better clarify actor effects, partner effects, and potential mediating pathways. This may help develop more precise and mechanism-informed dyadic nursing strategies for colorectal cancer postoperative recovery.

## 5 Conclusions

In this propensity score-matched retrospective cohort study, documented exposure to APIM-guided dyadic nursing was associated with more favorable postoperative recovery among colorectal cancer patient–caregiver dyads. Compared with usual postoperative nursing, APIM-guided dyadic nursing was linked to better patient-reported recovery and improved caregiver well-being in the matched cohort. Exploratory APIM analyses further suggested that self-efficacy and distress were important actor-related factors, while caregiver self-efficacy may have a modest partner association with patient recovery. These findings support the potential value of considering patients and caregivers as an interdependent care unit in postoperative nursing. However, because this was an observational retrospective study, the results should be interpreted as adjusted associations rather than definitive causal evidence. Prospective multicenter randomized controlled trials with standardized intervention delivery and longer follow-up are needed to confirm causality, sustainability, and implementation feasibility.

## Data Availability

The raw data supporting the conclusions of this article will be made available by the authors, without undue reservation.

## References

[1] Ferlay J Ervik M Lam F Colombet M Mery L Piñeros M . Global cancer observatory: cancer today. In: Lyon: International Agency for Research on Cancer. Lyon, France: International Agency for Research on Cancer (IARC), vol. 20182020. (2020). p. 557–67.

[2] Bray F Laversanne M Sung H Ferlay J Siegel RL Soerjomataram I . Global cancer statistics 2022: GLOBOCAN estimates of incidence and mortality worldwide for 36 cancers in 185 countries. Ca: A Cancer J For Clin. (2024) 74:229–63. doi: 10.3322/caac.21834 38572751

[3] Wu S Zhang Y Lin Z Wei M . Global burden of colorectal cancer in 2022 and projections to 2050: incidence and mortality estimates from GLOBOCAN. BMC Cancer. (2025) 25:1770. doi: 10.1186/s12885-025-15138-0 41239247 PMC12619147

[4] Sung H Siegel RL Laversanne M Jiang C Morgan E Zahwe M . Colorectal cancer incidence trends in younger versus older adults: an analysis of population-based cancer registry data. Lancet Oncol. (2025) 26:51–63. doi: 10.1016/s1470-2045(24)00600-4 39674189 PMC11695264

[5] Irani JL Hedrick TL Miller TE Lee L Steinhagen E Shogan BD . Clinical practice guidelines for enhanced recovery after colon and rectal surgery from the American Society of Colon and Rectal Surgeons and the Society of American Gastrointestinal and Endoscopic Surgeons. Dis Colon Rectum. (2023) 66:15–40. doi: 10.1097/dcr.0000000000002650 36515513 PMC9746347

[6] Vaz-Luis I Masiero M Cavaletti G Cervantes A Chlebowski RT Curigliano G . ESMO expert consensus statements on cancer survivorship: promoting high-quality survivorship care and research in Europe. Ann Oncol Off J Eur Soc For Med Oncol. (2022) 33:1119–33. doi: 10.1016/j.annonc.2022.07.1941 35963481

[7] Di Maio M Basch E Denis F Fallowfield LJ Ganz PA Howell D . The role of patient-reported outcome measures in the continuum of cancer clinical care: ESMO clinical practice guideline. Ann Oncol Off J Eur Soc For Med Oncol. (2022) 33:878–92. doi: 10.1016/j.annonc.2022.04.007 35462007

[8] Hardiman KM Felder SI Friedman G Migaly J Paquette IM Feingold DL . The American Society of Colon and Rectal Surgeons clinical practice guidelines for the surveillance and survivorship care of patients after curative treatment of colon and rectal cancer. Dis Colon Rectum. (2021) 64:517–33. doi: 10.1097/dcr.0000000000001984 33591043

[9] Husebø ALM Søreide JA Kørner H Storm M Wathne HB Richardson A . eHealth interventions to support colorectal cancer patients' self-management after discharge from surgery-an integrative literature review. Supportive Care Cancer: Off J Multinational Assoc Supportive Care Cancer. (2023) 32:11. doi: 10.1007/s00520-023-08191-7 PMC10700211 38055087

[10] Wang J Duan Y Geng L Li X Yue S Liu HJP . Trajectory of caregiver burden and associated factors in family caregivers of individuals with colorectal cancer: A longitudinal, observational multicenter study. (2024) 18:879–92. doi: 10.2147/ppa.s451487 PMC11033041 38645699

[11] Zhang M Xue Y Shao M Yang Y Yu L Ma B . The effects of a psychoeducational intervention on caregivers of colorectal cancer patients: a meta-analysis of randomized controlled trials. Eur J Oncol Nursing: Off J Eur Oncol Nurs Soc. (2025) 74:102739. doi: 10.1016/j.ejon.2024.102739 39729814

[12] Gong J Hu C Chen M Cao Q Li Q . Interventions to improve self-efficacy in colorectal cancer patients and/or caregivers: a systematic review and meta-analysis. J Oncol. (2021) 2021:4553613. doi: 10.1155/2021/4553613 34707659 PMC8545593

[13] Chen X Wang Z Zhou J Lin C Luo H Zhao J . The impact of self-perceived burden, caregiver burden, and dyadic coping on negative emotions in colorectal cancer patient-spousal caregiver dyads: a dyadic analysis. Front Psychol. (2023) 14:1238924. doi: 10.3389/fpsyg.2023.1238924 37818420 PMC10561240

[14] Qin F Wei T Zhao X Yuan S He Y Chen M . Relationship between family resilience and dyadic coping in colorectal cancer patients and their spouses, based on the actor-partner interdependence model. Eur J Oncol Nursing: Off J Eur Oncol Nurs Soc. (2024) 70:102622. doi: 10.1016/j.ejon.2024.102622 38795443

[15] Kim YM Kang NE Lee MR Ha GW Hong HC . Interdependence of health between patients with colorectal cancer and family caregivers: a cross-sectional study. BMC Nurs. (2025) 24:515. doi: 10.1186/s12912-025-03062-4 40355918 PMC12070582

[16] Jin Y Li X Ma H Xiong L Zhao M Wang H . Dyadic effects of perceived stress, relationship satisfaction and distress disclosure on emotional distress in colorectal cancer patients and their family caregivers: an actor-partner interdependence mediation model. Asia-Pacific J Oncol Nurs. (2024) 11:100580. doi: 10.1016/j.apjon.2024.100580 39351017 PMC11440261

[17] Iovino P De Maria M Corvese F Giordano V Alvaro R Vellone E . The influence of patient and caregiver depression on patient self-care and caregiver contribution to self-care in ostomy: a dyadic analysis. J Clin Nurs. (2023) 32:6441–9. doi: 10.1111/jocn.16676 36880219

[18] Iovino P De Maria M Corvese F Giordano V Alvaro R Vellone E . The influence of patient and caregiver depression on patient self‐care and caregiver contribution to self‐care in ostomy: a dyadic analysis. J Clin Nurs. (2023) 32:6441–9. doi: 10.1111/jocn.16676 36880219

[19] Kim YI Park IJ Ro JS Lee JL Kim CW Yoon YS . A randomized controlled trial of a digital lifestyle intervention involving postoperative patients with colorectal cancer. NPJ Digital Med. (2025) 8:296. doi: 10.1038/s41746-025-01716-w 40394118 PMC12092578

